# The complex relationship between microbial growth rate and yield and its implications for ecosystem processes

**DOI:** 10.3389/fmicb.2015.00615

**Published:** 2015-06-16

**Authors:** David A. Lipson

**Affiliations:** Department of Biology, San Diego State UniversitySan Diego, CA, USA

**Keywords:** bacterial growth efficiency (BGE), carbon use efficiency (CUE), climate change, copiotroph, growth rate, growth yield, microbial growth kinetics, oligotroph

## Introduction

Growth rate and efficiency are fundamental traits of microbes that significantly influence how communities and ecosystems function. However the microbiological literature shows an apparent contradiction in the relationship between growth rate and yield (defined as the portion of consumed substrate that is converted into biomass or ATP). Pirt (1965) defined maintenance energy in growing bacterial cultures and predicted that slow growth rates would be associated with inefficient growth. This idea is consistent with studies of bacterial growth efficiency (BGE) in marine and aquatic ecosystems. However, a separate body of literature supports a negative relationship between growth rate and yield, and finds that this rate-yield tradeoff is central in evolution and the coexistence of species. The concept of yield arises in the terrestrial biogeochemical literature, but under the term, carbon use efficiency (CUE), where researchers are concerned with the rates of carbon dioxide (CO_2_) lost from the ecosystem through microbial respiration, and how the partitioning of plant litter into soil carbon (C) and CO_2_ is affected by climate change. Here the rate-yield tradeoff also has important implications. I believe the seemingly contradictory points of view expressed in the literature can be reconciled by considering the growth conditions, ecological strategies and level of organization treated by these various studies. This opinion article is not intended as a comprehensive review. Excellent reviews and analyses of various aspects of this literature exist (Russell and Cook, 1995; Del Giorgio and Cole, 1998; Ferenci, 1999; Carlson et al., 2007; Van Bodegom, 2007; Wang and Post, 2012; Sinsabaugh et al., 2013). These works have resolved much of the perceived ambiguity in the field, but to my knowledge no one has directly addressed the apparent paradox mentioned above.

## Maintenance energy and its implications for growth yield

**Figure 1:**
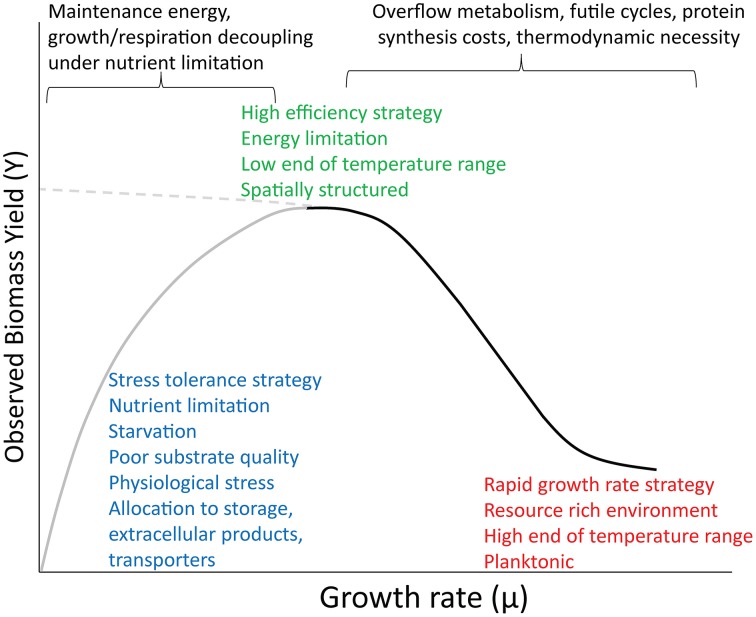
**Conceptual diagram showing a proposed relationship between growth rate and yield across a broad range of environmental conditions and ecological strategies**. The dominant forces structuring the two limbs of the curve appear at top. The left limb (gray) has a hyperbolic shape based on maintenance theory. The right limb (black) is sigmoidal, after Beardmore et al. (2011). The dashed line at left indicates the continuation of the simpler relationship between rate and yield that might be observed in the absence of these other phenomena.

Pirt (1965) differentiated between substrate used to produce new cells from that required to “maintain cells in a healthy state.” Assuming maintenance requirements are constant as the specific growth rate (μ) slows, a greater proportion of consumed substrate is used for maintenance and so the observed yield (Y) decreases (Pirt defines true growth yield, Y_G_, as the yield in the absence of maintenance costs. In this paper I deal mainly with Y, as this is what most studies measure). This results in a linear relationship in the double reciprocal plot (1/Y vs. 1/μ), which produces the hyperbolic relationship shown on the left side of Figure 1. Pirt provided several examples from continuous culture experiments that fit this expected relationship, and one example that produced a non-linear relationship. Even in 1965, Pirt acknowledged that maintenance requirements can shift with changes in factors such as aeration, pH and metabolic state, and used this logic to discount some earlier studies on maintenance (“qualitative” vs. “quantitative” changes in growth). Pirt (1987) later introduced a model of dormant vs. active cells to explain deviations from the expected yield at low growth rates, and other researchers have explicitly included metabolic state in their models (Panikov, 1996; Blagodatsky and Richter, 1998; Wang et al., 2014) It is now clear that maintenance varies with growth rate, and depends on a complex set of processes (Ferenci, 1999; Van Bodegom, 2007; Wang and Post, 2012). Van Bodegom (2007) lists the “non-growth” costs that influence the empirical measurement of maintenance (and therefore Y) as shifts in metabolic pathways, energy spilling reactions, motility, storage products, osmoregulation, extracellular losses, nucleic acid and protein turnover, and O_2_ stress responses. He points out that costs such as storage and extracellular losses should not be strictly considered part of physiological maintenance (or endogenous metabolism), and that cell death can also contribute to Y. Wang and Post (2012) reconcile three models of maintenance, one of which (Herbert) includes a term that can represent cell death. All of these variants predict decreased Y at low growth rates and substrate concentrations.

Researchers in nutrient-limited aquatic ecosystems have noted positive relationships between bacterial growth rate and BGE (defined as bacterial production divided by the sum of production and respiration). Discounting other fates of consumed substrate, namely extracellular products, BGE is analogous to Y. Low BGE are observed under oligotrophic conditions (Del Giorgio and Cole, 1998; Smith and Prairie, 2004). It is argued that these planktonic oligotrophs maintain energetically expensive processes that allow them to maximize growth rate rather than efficiency (Westerhoff et al., 1983; Carlson et al., 2007). This strategy makes sense when growth is limited by mineral nutrients rather than by organic C (Smith and Prairie, 2004; Carlson et al., 2007), since excess C can be invested in foraging for increased nutrient uptake (transporters, chelators, motility, etc.) (Lauro et al., 2009). Note that by more specific and modern definitions, not all of these factors are considered maintenance costs (Van Bodegom, 2007). This inefficient strategy should also be more successful in spatially non-structured, planktonic environments, compared to highly structured environments such as biofilms or soils (Kreft, 2004). However, spatial heterogeneity can also play a role in structuring microbial strategies in marine and aquatic habitats (Arnosti et al., 2013).

## Rate-yield tradeoffs in growth

There is considerable theoretical and empirical support for a negative relationship between growth rate and yield in microbial metabolism. A tradeoff between rate and efficiency is intuitive in the mechanical analogy: hot rods and monster trucks get lower gas mileage than slower, less powerful, fuel-efficient models. However, the physiological basis for this tradeoff in microbes is less obvious. Some authors (Westerhoff et al., 1983; Pfeiffer et al., 2001) offer a thermodynamic explanation: for a reaction to be 100% efficient, the energy of the products would equal that of the reactants, and so the rate would be zero; a decreased ATP yield with energy lost as heat could speed up the reaction at the expense of efficiency. A more tangible biochemical perspective is that at rapid growth rates where anabolism and catabolism become unbalanced, energy is dissipated through energy-spilling reactions involving futile cycles (Russell and Cook, 1995) or overflow metabolism (secretion of excess metabolites) (Carlson et al., 2007). Additionally, because of the high energetic cost of producing protein, higher rates of protein synthesis in fast-growing cells can lead to lower efficiency relative to a state with lower growth rate in which yield is maximized (Molenaar et al., 2009; Wong et al., 2009). For this reason, amino acid costs are minimized in highly expressed and secreted proteins (Akashi and Gojobori, 2002; Smith and Chapman, 2010). A review of the metabolic basis and evidence for rate-yield tradeoffs can be found in the Supplemental Information (SI) of Beardmore et al. (2011). This tradeoff tends to create two divergent ecological strategies: fast-growing but inefficient vs. slow-growing but efficient. The selection of these strategies can depend on resource availability: rapid growth is expensive (more transporters, enzymes, and overflow metabolism) and only used under high resource conditions, whereas the metabolically efficient, high yield strategy is employed where resources are scarce (Molenaar et al., 2009). Others show that the high yield strategy is a hallmark of cooperative populations, and these can be particularly successful in spatially structured environments where they are insulated from fast-growing, competitive neighbors (Pfeiffer et al., 2001; Kreft, 2004; Kreft and Bonhoeffer, 2005). However, the rate-yield tradeoff can lead to coexistence of two strategies even in a well-mixed environment, given that the slow-growing strategy exists in a flatter fitness landscape (for example, having slower mutation rates) (Beardmore et al., 2011).

### Rate-yield tradeoffs within single species

There are several examples of microbes shifting between high yield/low rate and the converse state depending on growth conditions (see references in Kreft, 2004). Novak et al. (2006) found rate-yield tradeoffs within individual populations of *E. coli*, though not among different populations. The classic work of Monod (1942) shows a negative relationship between growth rate and cellular yield of *E. coli*, with yield decreasing and rate increasing with increasing temperature (see SI of Beardmore et al., 2011). Similar relationships among rate, yield and temperature can be seen in the growth of *Cobetia marina* (Figure 1 in Yumoto et al., 2004) and in an *Arthrobacter* species from Arctic soil (Figure 4A in Panikov and Sizova, 2007). The rate-yield tradeoff observed with changes in temperature implies a thermodynamic basis, as discussed earlier. According to the Arrhenius equation, the reaction rate increases with temperature. As rates increase, there might be more decoupling from ATP production. This is consistent with studies of warming and CUE (discussed below).

### Rate-yield tradeoffs within communities

Negative relationships between growth rate and yield are sometimes found among species and within communities. In experiments on soils incubated with substrate, a rate-yield tradeoff was observed across seasonal gradients and among samples that varied in fungal: bacterial ratio (Lipson et al., 2009). Two opposing strategies were also found among sulfur-oxidizing bacteria from soda lakes (Sorokin et al., 2003), and there is evidence for a rate-yield tradeoff among yeast species (Weusthuis et al., 1994).

The form of rate-yield tradeoffs is sometimes observed to be linear (Lipson et al., 2009), but a case has been made for a sigmoidal form of this relationship (Beardmore et al., 2011), resulting from a shift between two distinct states (Kappler et al., 1997). This shape is depicted on the right half of Figure 1.

## Reconciling tradeoffs and maintenance

Despite the wide variety of contexts in which the preceding theories and data were developed, there is no reason they cannot be reconciled under a broader perspective. Positive relationships between growth rate and yield are observed in oligotropic aquatic environments and in chemostats at low dilution rates. Rate-yield tradeoffs are observed in richer growth media, soils and other environments varying in resource availability, and between planktonic and spatially structured environments. I propose that all of these observations are part of the same continuum (Figure 1). The overall shape of this curve is consistent with the “hidden square root boundary” of Wong et al. (2009), in which the maximum growth rate is limited to the square root of the product of yield, substrate turnover number, and the maximum synthesis rate of the transporter or turnover enzyme. The positive relationship posited by Pirt pertains to conditions where growth rate is highly constrained by nutrient limitation or physiological stress, such as super or suboptimal pH (Koussémon et al., 2003), superoptimal temperatures (Monod, 1942), and extreme cases of energy limitation (near-starvation conditions). In addition to low substrate quantity, low yields can also arise from poor substrate quality (Westerhoff et al., 1983; Schmidt et al., 2004). This positive relationship can be mediated by physiological maintenance, but also by other non-growth costs, such as allocation to extracellular enzymes, chelators, slime, etc. For example, the social myxobacterium, *Sorangium cellulosum*, grows slowly and has a very low apparent yield, partly owing to its large investment in lipids and secondary signaling compounds (Bolten and Muller, 2009). The positive correlation observed between production rate and BGE is indirect, and arises when both BGE and production rate are limited by nutrients such as P, as growth and respiration become decoupled. At intermediate and higher levels of resource availability, the rate-yield tradeoff becomes significant. Here we see the dichotomy of slow-growing, efficient vs. fast-growing, wasteful strategies seen along resource gradients and between spatially structured vs. planktonic microhabitats (Pfeiffer et al., 2001; Kreft, 2004; Costa et al., 2006; Frank, 2010), and also the variation in rate and yield seen in microbes growing in rich media over a wide temperature range (discussed above).

## Ecosystem implications for rate-yield relationships

There is a growing effort to incorporate microbial growth kinetics into larger scale ecosystem studies (Schimel and Weintraub, 2003; Monson et al., 2006; Lipson et al., 2009; Treseder et al., 2012; McCalley et al., 2014), and concepts of growth efficiency have also been used to unite metabolic theory of ecology and ecological stoichiometry theory (Frost et al., 2006; Allen and Gillooly, 2009). CUE is increasingly incorporated into soil C models (Allison et al., 2010; Cotrufo et al., 2013; Sinsabaugh et al., 2013; Wieder et al., 2014). In the aquatic literature, BGE is observed to decline with increasing temperature (Apple et al., 2006), though some argue that this effect is mediated by nutrient availability (López-Urrutia and Morán, 2007). A similar debate exists in the terrestrial biogeochemistry literature: short-term reductions in CUE with warming have been reported (Steinweg et al., 2008; Manzoni et al., 2012), but in the long term, microbial communities may adapt (Bradford et al., 2008; Frey et al., 2013; Tucker et al., 2013). However, the rate-yield tradeoff can limit the extent and impact of CUE adaptation to temperature (Allison, 2014). As seen in aquatic ecosystems, the impacts of mineral nutrients can also have an overriding effect on CUE in soils (Keiblinger et al., 2010; Manzoni et al., 2012). The uncertainty in the literature emphasizes the need to understand the controls over microbial growth yield, how the ecological strategies defined by growth rate and yield will be affected by climate change and how these microbial characteristics will feed back to influence ecosystem respiration and C sequestration in soils. A clearer understanding of the relationship between growth rate and yield under a wide range of conditions will be necessary to meet these goals.

### Conflict of interest statement

The author declares that the research was conducted in the absence of any commercial or financial relationships that could be construed as a potential conflict of interest.
